# FRAILTY TRAJECTORIES AND ITS ASSOCIATED FACTORS IN JAPANESE OLDER ADULTS

**DOI:** 10.1093/geroni/igae098.2762

**Published:** 2024-12-31

**Authors:** Yu Taniguchi, Akihiko Kitamura, Toshiki Hata, Koji Fujita, Takumi Abe, Yu Nofuji, Satoshi Seino, Yuri Yokoyama

**Affiliations:** National Institute for Environmental Studies, Tsukuba, Ibaraki, Japan; Tokyo Metropolitan Institute for Geriatrics and Gerontology, Tokyo, Japan; Tokyo Metropolitan Institute for Geriatrics and Gerontology, Tokyo, Japan; Tokyo Metropolitan Institute for Geriatrics and Gerontology, Tokyo, Japan; Tokyo Metropolitan Institute for Geriatrics and Gerontology, Tokyo, Japan; Tokyo Metropolitan Institute for Geriatrics and Gerontology, Tokyo, Japan; Tokyo Metropolitan Institute for Geriatrics and Gerontology, Tokyo, Japan; Tokyo Metropolitan Institute for Geriatrics and Gerontology, Tokyo, Japan

## Abstract

The present study had two objectives: to identify aging trajectories in frailty status from age 65–90 years among community-dwelling older Japanese; and to identify factors associated with this trajectory among these people. The present prospective study used repeated measures data on frailty status from a 13-year longitudinal study. 1706 older adults completed an annual frailty assessment at least once during the follow-up. Frailty status was determined using an index based on the Fried frailty phenotype criteria. Potential associated factors for frailty trajectory included physical, biological, lifestyle-related, and psychological factors, as well as comorbidities. We identified five trajectory patterns in the frailty score from age of 65 to 90 years —individuals who were robust (10.5%) as well as individuals with late-onset frailty (16.1%), middle-onset frailty (25.6% and 35.2%), and early-onset frailty (12.7%). The early-onset frailty group were already pre-frail at the age of 65 years, and their frailty score gradually progressed to frail status. Compared with the robust group, the early-onset group showed a higher prevalence of cerebrovascular diseases, bone and joint diseases, poor nutrition, sarcopenia, hospitalization, low cognitive function, and smoking at the end of follow-up. Associated factors in the middle-onset group largely overlapped with those of the early-onset group. The late-onset frailty group tended to have a higher association with heart disease and bone and joint diseases compared with the robust group. Proposed effective population-based frailty prevention strategies in each age group may contribute to effective strategies to extend healthy life expectancy.

